# Gender differences in response to war-related trauma and posttraumatic stress disorder – a study among the Congolese refugees in Uganda

**DOI:** 10.1186/s12888-019-2420-0

**Published:** 2020-01-10

**Authors:** Herbert E. Ainamani, Thomas Elbert, David Kani Olema, Tobias Hecker

**Affiliations:** 1 Division of Health Psychology and Psychiatry, Kabale University School of Medicine, P.O.BOX 317, Kabale, Uganda; 2 Department of Psychology, Faculty of Health Sciences and Nursing Bishop Stuart University, Mbarara, Uganda; 3 Department of Psychiatry, Mbarara University of Science and Technology, Mbarara, Uganda; 4 Department of Psychology, University of Konstanz, Konstanz, Germany; 5 Department of Education Science, Busitema, Tororo, Uganda; 6 Department of Psychology, Bielefeld University, Bielefeld, Germany

**Keywords:** Refugees, Gender, War, Trauma, Posttraumatic stress disorder (PTSD)

## Abstract

**Background:**

The wars in the Democratic Republic of Congo have left indelible marks on the mental health and functioning of the Congolese civilians that sought refuge in Uganda. Even though it is clear that civilians who are exposed to potentially traumatizing events in war and conflict areas develop trauma-related mental health problems, scholarly information on gender differences on exposure to different war-related traumatic events, their conditional risks to developing PTSD and whether the cumulative exposure to traumatic events affects men and women differently is still scanty.

**Methods:**

In total, 325 (*n* = 143 males, *n* = 182 females) Congolese refugees who lived in Nakivale, a refugee settlement in the Southwestern part of Uganda were interviewed within a year after their arrival. Assessment included exposure to war-related traumatic events, and DSM-IV PTSD symptom severity.

**Results:**

Our main findings were that refugees were highly exposed to war-related traumatic events with experiencing dangerous flight as the most common event for both men (97%) and women (97%). The overall high prevalence of PTSD differed among women (94%) and men (84%). The highest conditional prevalence of PTSD in women was associated with experiencing rape. The dose-response effect differed significantly between men and women with women showing higher PTSD symptom severity when experiencing low and moderate levels of potentially traumatizing event types.

**Conclusion:**

In conflict areas**,** civilians are highly exposed to different types of war-related traumatic events that expose them to high levels of PTSD symptoms, particularly women. Interventions focused at reducing mental health problems resulting from war should take the context of gender into consideration.

## Background

The Democratic Republic of Congo (DRC) has had a long history of unrest and instability which has exposed the citizens to severe traumatic experiences such as war atrocities, physical torture, mass execution, disappearances and rape that carry insurmountable psychological effects [1]. Studies with refugees from the DRC have revealed that many of them witnessed their fellow civilians being killed, kidnapped, sexually abused, and enslaved [2–4]. Correspondingly, a study among refugees [5] reported that 73% of the participants from DRC who lived in Nakivale at the time of assessment had witnessed dead or mutilated bodies, 69% shelling or bomb attacks, 68% were injured with a weapon, 60% reported witnessing burning houses and 60% experienced crossfire or sniper attacks. Even though gender estimates are not clear in this study, the authors revealed that female refugees experienced more potentially traumatizing events than their male counterparts.

Epidemiological studies on trauma have continually indicated that men and women differ not only in their risk of being exposed to different traumatic events, e.g. with more women likely to experience conflict-related sexual violence than men [6, 7] but also in subsequent development of posttraumatic stress disorder [6–9]. Previous research examining gender differences in trauma spectrum disorders including PTSD have always reported a significant gender difference with women reporting higher symptom severity [10, 11]. Also, a study that compared gender differences in PTSD according to DSM-5, among earthquake survivors showed that female participants had higher PTSD prevalence and symptom severity in almost all DSM-5 PTSD symptom clusters [12].

Prior research has suggested the potentially traumatizing event with the highest conditional risk of developing PTSD to be rape among women and combat exposure among men [13]. This corresponds well with other findings that showed women not to only have a higher likelihood of experiencing sexual violence in war settings but also to have a higher risk for developing PTSD than men [8, 14]. Other researchers have argued that sexual violence with its high conditional risks for PTSD among women may explain gender differences in the overall prevalence of PTSD [15, 16]. However, in a study among refugees in Nakivale refugee settlement, it was suggested that gender differences in the prevalence of PTSD may arise from exposure to the overall trauma load as women were more likely than men to experience devastating events such as sexual violence [5]. This hypothesis was also supported by findings from a meta-analysis on 18 studies that showed an overall gender difference in PTSD with females being more vulnerable to PTSD than men [17] . However, other scholars [18] argue that gender differences in PTSD cannot be explained fully by the traumatic event types that have been experienced; thus, supporting the fact that the role of gender in the development or maintenance of PTSD deserves more attention.

In line with this, it has been argued that even though estimates of PTSD prevalence vary between men and women, PTSD symptoms severity increases with trauma load, i.e., the number of traumatic experiences, in the general population. This is also referred to as dose-response effect or *building block effect* [4, 19–21]. The main difference between the dose-response effect and the building block effect is that following the building block idea each potentially traumatizing event type is counted but the frequency of events is not considered. Each traumatic event type like a “building block” adds up to the vulnerability of mental illness, particularly of PTSD, among the survivors of trauma [22]. Although this may explain the variation of PTSD rates across different postwar communities, gender differences in the *building block effect* have not yet been investigated in survivors of war.

On the other hand, scholars argue that different types of potentially traumatizing events differ in their potential of causing the development of PTSD [16, 23]. To further investigate this, our study aimed at examining exposure to war-related potentially traumatizing events and their associated conditional risks of developing PTSD among the Congolese refugees in Uganda. Furthermore, we examined gender differences in PTSD symptom severity in response to cumulative exposure to potentially traumatizing event types. We hypothesized that (1a) male refugees would experience more different types of war- related potentially traumatizing events than females. (1b) We postulated that female refugees would report the experience of sexual violence more often than males. (2) We hypothesized that exposure to sexual violence would have a high conditional risk for the development of PTSD among the female refugees. (3) If so, we expected that women would show a steeper building block effect than men.

## Methods

### Participants

The study was conducted among the Congolese refugees in Nakivale Refugee Settlement in Insingiro District-southwestern Uganda between the months of March and June 2013. At the time of assessment, this settlement had just received a high influx of refugees from the DRC. In this study, only Congolese refugees who had arrived after 2012 and had stayed in the settlement for at least a period of 1 month prior to the day of the interview were included. This inclusion criterion was validated at the beginning of each interview. It was established in order to make sure that only those refugees who had fled from the DRC as a consequence of the then most recent wave of conflicts between the DRC army and M23 rebels were surveyed. Although, there were many other Congolese refugees who had stayed in the settlement for many years up to decades, they were not included in this study since we were only interested in the consequences of the then recent experiences of war and violence. Of the 325 included refugees, 182 (56%) were female (mean age: 30.9 years) and 141 were male (mean age: 31.9 year). The age of the total sample ranged between 18 and 65 years. All participants reported to have fled from Eastern DRC because of the then prevailing war violence (for more details on the sample see Table 1).

**Table 1:** Demographic characteristics, PTSD diagnosis and trauma load in relation to gender

	Without PTSD				With PTSD				
	Male		Female	Sex difference	Male	Female		Sex difference	
	23(16.3)		11(6.2)		118(83.7)	167(93.8)			
Demographics	n (%)		n (%)	× ^2^	n (%)	n (%)		× ^2^	
Education									
No education	3(13.0)		6(54.5)	13.44**	16(13.8)		53(32.1)	16.1**	
Primary	11(47.8)		0(0)		48(41.4)		47(28.5)		
High school	5(21.7)		5(45.5)		49(38.8)		62(37.6)		
College	4(17.4)		0(0)		7(6.0)		3(1.8)		
Tribe									
Hutu	18(78.3)		9(81.8)	1.25	86(72.9)		103(61.3)	4.59	
Nyamurenge	2(8.7)		0(0)		19(16.1)		32(19.2)		
Nandi	1(4.3)		1(9.1)		7(5.9)		18(10.8)		
Hunde	2(8.7)		1(9.1)		6(5.1)		14(8.4)		
	Males					Females			
	N	M	SD			N	M	SD	t
Age	141	31.93	9.03			182	30.9	9.01	1.06
PTSD Symptoms	141	29.71	13.89			179	35.70	11.64	−4.19**
Trauma load	141	18.50	3.84			182	18.96	3.94	−1.05

Correlation is significant at *p < .05, **p < .01, ***p < .001

### Procedure

Ethical clearance to conduct research was obtained from Mbarara University of Science and Technology Research Ethics Committee (MUST-REC) and Uganda National Council for Science and Technology (UNSCT). Further permission was sought from the Office of the Prime Minster (OPM), a department of the government of Uganda that is charged with overseeing the plight of the refugees within the country.

One social scientist from the Congolese refugee community and three psychologists who were trained in psychological assessments and interview skills conducted the semi-structured interviews in Kiswahili and Kinyabwisha (two languages spoken by the Congolese refugees living in Nakivale refugee settlement). During training, the interviewers practiced the assessment in form of role plays to ascertain high inter-rater reliability.

Through leaders of different villages within the refugee settlement, we were able to identify the Congolese refugees that had arrived after 2012**.** Due to the nature of our sample (i.e., recently arrived refugees), we used a snowball sampling technique to recruit the participants who were living within the villages that accommodated the Congolese refugees at the time of our data collection. Following ethical considerations, the objectives of the study were fully explained to all the study participants before a written informed consent was signed. Participants who did not know how to read and write consented with their fingerprints instead of signature. Face to face interviews were conducted in a highly conducive environment without any disruption. At the end of each interview, participants were given a bar of soap and a package of salt as compensation for their time.

### Measures

Our semi-structured interviews included information related to social socio-demographic (e.g. age, gender, educational background.

Exposure to war-related trauma was assessed using a checklist of 25 war-related traumatic-event types that included aspects like; having experienced or witnessing torture, abduction, physical or sexual assault, rape by armed personnel, seeing dead bodies or mutilations and being wounded by a weapon. This was an adapted version of a checklist that had been previously used by Neuner and colleagues in a comparable sample [19] and that had shown high test-retest reliability (*r* = .73, *p* < .001) and rhymed with the composite international diagnostic interview in comparable samples in Uganda and other war-affected communities [24–27]. This checklist has also been successfully used in studies within the DRC [24] and Congolese refugees in Nakivale refugee settlement [27, 28]. Generally, trauma exposure was represented by adding up the total number of traumatic event types experienced by each participant (range: 0–25) as recommended by previous researchers [21].

PTSD symptoms severity was assessed using PTSD Symptom Scale–Interview (PSS-I). The 17 DSM-IV symptom criteria for PTSD are assessed with one question for each symptom and refer to the previous 2 weeks [29]. The answers were coded on a 4-point scale ranging from not at all (0) to five or more times per week/very much (3). The PSS-I has been shown to have good psychometric properties (e.g., Cronbach’s α = .86, inter-rater reliability = .93 [30];. It has also been validated for use in Uganda [24] and has been successfully used in the DRC [31] and with Congolese refugees in Nakivale refugee camp [27, 28]. We computed a total sum score for the PTSD symptom severity by adding all item scores. Cronbach’s alpha coefficient of the total score was .96 in the present sample.

### Data analysis

Statistical analyses were performed in SPSS Statistics 22 for Mac (SPSS Inc., Chicago, IL). Chi-square tests and independent t-tests were used to examine gender differences in demographic characteristics, war-related potentially traumatizing event types and PTSD diagnosis and symptom severity. Pearson correlation coefficient was used to analyze associations between the number of war-related potentially traumatizing event types and PTSD symptoms severity in the total sample and in men and women separately. Following the study of Neuner and others [19], we analyzed the effect of cumulative exposure in relation to PTSD symptom severity by dividing the sample in different sub-groups based on the number of war-related potentially traumatic event types reported, i.e. in the sense of the building block which counts types but does not weigh frequency (for justification see [19, 20]). We divided the sub-groups as follow: very low (0–4), low (5–8), moderate (9–12), high (13–16), very high (17–20) and extremely high (21–24) war-related potentially traumatizing event types. A two-way Analysis of Variance was used to analyze group differences and the interaction effects between PTSD symptoms severity, gender and sub-group levels of trauma exposure. Missing values were excluded listwise. No variable deviated significantly from normal distribution and variance–covariance matrices showed homogeneity. Level of significance was set to an alpha of .05 and analyses were calculated two-tailed. Effect size η^2^ ≥ 0.01, η^2^ ≥ 0.06 and η^2^ ≥ 0.14 was considered to represent a small, moderate and large effects correspondingly.

## Results

### Prevalence of war-related traumatizing event types

In total, almost all participants in our sample had experienced at least one potentially traumatizing event. Overall, there was no significant difference between male and female refugees regarding their exposure to the number of potentially traumatizing event types (*see* Table 1). However, male participants reported having been imprisoned significantly more often than females (*χ*^*2*^ = 12.62, *df* = 1, *p* = < .001). In contrast, there was a significant gender difference regarding witnessing of rape by armed soldiers: females reported to have witnessed the event more often than male respondents (*χ*^*2*^ = 20.18, *df* = 1, *p = < .001*). A corresponding gender difference was obtained for being raped (*χ*^*2*^ = 57.06, *df* = 1, *p* < .001) with women reporting being raped significantly more often than men. Details are presented in Table 2.

**Table 2:** Prevalence of war-related traumatic events among the Congolese refugees

	Total		Male		Female		
	*n* = 325		*n* = 143		*n* = 182		Gender difference
Event	*n*	%	*n*	%	*n*	%	χ^2^
Witnessed harassment by soldiers	314	97.2	139	98.6	175	96.2	1.73
Experiencing dangerous flight	313	96.9	137	97.2	176	96.7	.06
Witnessing looting by soldiers	311	96.3	137	97.2	174	95.6	0.54
Seeing dead bodies	311	96.3	136	96.5	175	96.2	0.02
Close to crossfire/shootings	309	95.7	133	94.3	176	96.7	1.08
Seeing someone being injured by a weapon	308	95.4	132	93.6	176	96.7	1.71
Experiencing torture by soldiers	306	94.7	135	95.7	171	94	0.51
Experienced harassment by soldiers	290	89.8	129	91.5	161	88.5	0.79
Close to burning houses	287	88.9	131	92.9	156	85.7	4.15**
Being victim of robbery by soldiers	281	87	123	87.2	158	86.8	0.01
Forced to pay taxes by soldiers	275	85.1	125	88.7	150	82.4	2.44
Abduction witnessed	275	85.1	113	80.1	162	89	4.49**
Close to grenade/bomb attack	263	81.4	116	82.3	147	80.8	0.12
Seeing someone being tortured by soldiers	247	76.5	120	85.1	127	69.8	10.37**
Witnessing murder	242	74.9	100	70.9	142	78	2.13
Sexual assault experienced	216	66.9	83	58.9	133	73.1	7.24*
Witnessed sexual assault	210	65	81	57.4	129	70.9	6.30**
Witnessing rape of a woman by soldiers	196	60.7	66	46.8	130	71.4	20.18***
Having been imprisoned	136	42.1	75	53.2	61	33.5	12.62***
Being injured by a weapon	131	40.6	61	43.3	70	38.5	0.76
Experiencing rape	123	38.1	21	14.9	102	56	57.06***
Abduction experienced	94	29.1	48	34	46	25.3	2.9
Fighting in the combat	31	9.6	20	14.2	11	6.0	6.07**

Correlation is significanr at , *p < .05, **p < .01, *** *p* < .001, Pearson Chi-square test x^2^

### PTSD prevalence

Generally, 285 participants (89%) of our entire sample fulfilled the DSM-IV criteria for PTSD diagnosis. More women (*n* = 167; 93.8%) than men (*n* = 118; 83.7%) presented with PTSD (χ^2^ = 8.48, *df* = 1, *p* < .001). Additionally, our results revealed that female participants presented with higher PTSD symptoms severity than men (see Table 1).

### Conditional risk of PTSD prevalence

In this study, we examined the conditional risk of PTSD prevalence following the self-reported worst experienced traumatic event. Results indicated the highest conditional risk of the prevalence of PTSD to be associated with *experiencing rape* for women (97% of *n* = 70) and men (100% of *n* = 2). This was followed by witnessing murder with 96% of *n* = 49 for women and 91% of *n* = 42 for men. Witnessing people carrying dead bodies emerged as the third highest conditional risk of PTSD in women (89% of *n* = 16) and men (69% of *n* = 9). More details are presented in Table 3.

**Table 3:** Conditional prevalence of PTSD on the worst traumatic event

	Subjective worst traumatic experiences		Conditional Prevalence of PTSD	
	Males	Females	Males	Females
Worst traumatic events endorsed by the participants	N (%)	N (%)	N (%)	N (%)
Experiencing a natural disaster	0(0.0)	1(0.6)	0(0.0)	1(100)
Experiencing a serious accident	1(0.7)	0(0.0)	0(0.0)	0(0.0)
Suffering from a life-threatening illness	0(0.0)	2(1.1)	0(0.0)	2(100)
Being close to a combat situation	1(0.7)	3(1.7)	1(100)	3(100)
Being close to crossfire or shootings	3(2.2)	5(2.8)	1(33.3)	4(80)
Being close to a bomb or grenade attack	1(0.7)	0(0.0)	0(0.0)	0(0.0)
Experiencing a dangerous flight	0(0.0)	1(0.6)	0(0.0)	1(100)
Being deprived of food	0(0.0)	2(1.1)	0(0.0)	1(50)
Being harassed by armed personnel	5(3.6)	4(2.2)	4(80)	3(75)
Being forced to pay taxes by armed personnel	1(0.7)	0(0.0)	1(100)	0(0.0)
Being a victim of robbery or looting by armed personnel	15(10.8)	9(5.1)	10(66.7)	7(87.5)
Witnessing torture of others by armed personnel	2(1.4)	0(0.0)	2(100)	0(0.0)
Being severely tortured by armed personnel	19(13.7)	0(0.0)	2(100)	0(0.0)
Seeing someone who was severely injured by armed personnel	1(0.7)	1(0.6)	1(100)	1(100)
Being injured by a weapon or a gun	10(7.2)	3(1.7)	9(90)	2(66.7)
Witnessing someone who was abducted	4(2.9)	3(1.7)	4(100)	3(100)
Being abducted by force	11(7.9)	1(0.6)	10(90.9)	1(100)
Witnessing rape	2(1.4)	0(0.0)	2(100)	0(0.0)
Experiencing Sexually assault	0(0.0)	1(0.6)	0(0.0)	1(100)
Being raped	2(1.4)	71(40.4)	2(100.0)	70(97.2)
Seeing People with dead bodies	13(9.4)	18(10.1)	9(69.2)	16(88.9)
Witnessing a killing or murder of someone	46(33.1)	51(28.7)	42(91.3)	49(96.1)
Fighting in a combat	2(1.4)	0(0.0)	2(100)	0(0.0)
Being socially excluded by a family member	0(0.0)	1(0.6)	0(0.0)	1(100)
Being assaulted by a weapon from a family member	1(0.7)	1(0.5)	1(100)	0(0.0)
Being sexually assaulted by a community member	1(0.7)	0(0.0)	1(100)	0(0.0)

### Gender differences in the building block effect

We found an association between the number of potentially traumatizing event types and PTSD symptom severity in our total sample (*r* = .59*, p* = < .001*)*. This positive correlation could be detected in both male (*r =* .50*, p = < .*001*)* and female participants (*r* = .70*, p* < .001). However, it was significantly stronger in female participants (*z* = 2.81, *p* = .002).

**Fig. 1:**
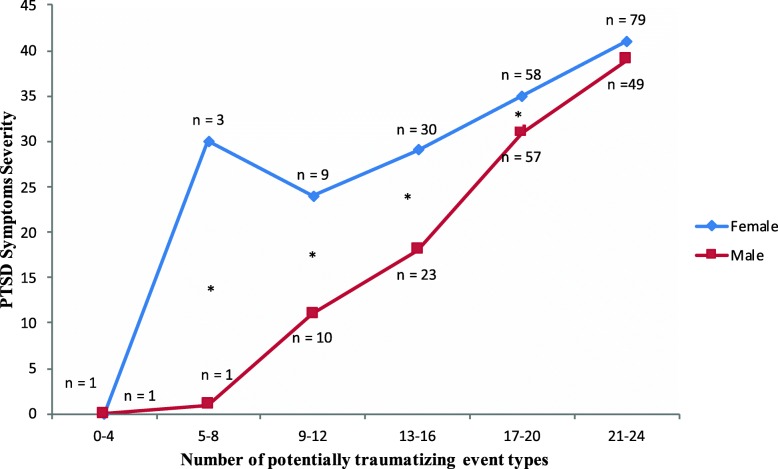
The building block effect separated for male and female refugee: PTSD symptoms severity in relation to the number of experienced war-related potentially traumatizing event types

A two-way ANOVA revealed a statistically significant interaction between the number of potentially traumatizing event types and gender on PTSD symptoms severity (*F* (5, 308) = 2.67, *p* < .05, η^2^ = .036,). Simple main effect indicated that women showed higher PTSD symptom severity than men within the sub-groups of low, moderate and high levels of traumatic event types. There were no gender differences in the sub-group of extremely high level of traumatic event types (see Table 4 and Fig. 1 for the observed gender differences in PTSD in relation to the cumulative exposure to different traumatic events).

**Table 4:** Gender differences in the PSS-I total scores across different levels of trauma exposure

Trauma Load	Female	Male			
	M (SD)	M (SD)	Mean difference	Std Error	*P*
Very low trauma (0–4)	0 (−)	0 (−)	< .01	14.37	> .99
Low trauma (5–8)	29.67 (17.01)	1.00 (−)	28.68	11.73	.015
Moderate trauma (9–12)	21.38 (16.04)	11.00 (8.62)	10.38	4.82	.032
High trauma (13–16)	28.67 (12.40)	17.96 (8.01)	10.71	2.82	<.001
Very high trauma (17–20)	35.47 (10.44)	30.54 (12.92)	4.92	1.90	.010
Extremely high trauma (21–24)	40.67 (7.84)	39.27 (7.48)	1.41	1.85	.447

### Post-hoc analysis: being raped and PTSD diagnosis and symptom severity

The experience of being raped showed to have the highest conditional risk of developing PTSD in our sample. However, women were much more likely to report that they have been raped. Therefore, in post-hoc analysis, we compared male and female refugees who reported that they had been raped with males and females who did not report to have been raped in regard to their PTSD symptom severity and PTSD diagnosis. We found that the four groups differed significantly concerning their PTSD symptom severity (*F* (3, 317) = 19.36, *p* < .001). In a Bonferroni-adjusted post-hoc analysis, female refugees who had been raped (*n* = 102, *M* = 39.66, *SD* = 8.30) reported higher PTSD symptom severity than female refugees (*n* = 78, *M* = 30.63, *SD* = 13.31, *p* < .001) and male refugees (*n* = 120, *M* = 28.18, *SD* = 13.80, *p* < .001), who had not been raped. However, they did not differ significantly from male refugees who had been raped (*n* = 21, *M* = 38.48, *SD* = 11.17, *p* > .999). Male refugees who had been raped showed higher PTSD symptom severity than females (*p* = .050) and male refugees (*p* = .002) who had not been raped. Male and female refugees who had not been raped did not differ in their PTSD symptom severity (*p* = .967). We found a very similar pattern in regard to PTSD diagnosis and the highest prevalence with 99% was shown by women who had been raped, followed by men who had been raped (91%). Nevertheless, we found high prevalence of PTSD rates among both female (87%) and male refugees (83%) who had not been raped.

## Discussion

The present study focused on gender differences in the profile of war-related potentially traumatizing event types, the conditional risk for the development of PTSD and on gender variation within the building block effect. Contrary to our hypothesis 1a, we found that in general, there were no differences between men and women regarding exposure to the number of war-related traumatic event types. At the same time, however, our results showed that (1b) Female refuges reported experiences of sexual violence than men (2) Female refugees with a comparable trauma load reported a higher PTSD symptom severity and a higher likelihood of presenting with PTSD than men.

Our results indicate that this sample of Congolese refugees that were living in Nakivale refugee settlement in western Uganda had experienced many and severe traumatic events and consequently presented with high PTSD prevalence. Specific potentially traumatizing event types that this sample was exposed to included *being close to combat situations*, *seeing someone being injured by a weapon* and *being harassed by armed personnel*. This high exposure to different war-related events may be explained by the prolonged war in DRC and the presence of different armed groups that have ravaged the country within the last two decades.

Consistent with Hypothesis 1b, the rates of rape as a main driver of trauma-related disorders was much higher in women than men. It seems questionable however, if a comparison for rape between men and women is meaningful, given their different biological alarm signals. For example, in war and post conflict settings, men struggle with the loss of their social status while women may struggle with not having the opportunity to choose fathers of their offsprings. Moreover, the violent nature related to acts of sexual violence and to aggressive proximity of the perpetrator is likely to involve responses such as dissociations, avoidance, numbing, shame and guilt [32–35]. It is worth noting that there was a substantial number of male participants who reported being raped (*n* = 21; 15%) and this subgroup reported enhanced levels of PTSD symptom severity and higher PTSD prevalence, just as women who reported that they had been raped. Out of the total number of male participants who experienced rape, only two endorsed exposure to rape as their worst traumatic event. The conditional risk of developing PTSD among male participants in this sample was 100% (though it was based on these two cases only).

The observation that women have been exposed to higher levels of sexual violence than men supported prior findings that generally showed women to have been more frequently exposed to sexual violence in war and post-conflict communities [5, 36]. In consonance with prior studies on refugee mental health [2, 5], our findings showed that women were more likely to suffer from PTSD than men. This corresponds well with findings of a study on gender differences in symptoms of PTSD after exposure to a terror attack [37]. In this previous study, women reported higher levels of symptoms on all subscales of PTSD symptoms than men. What is unique is that the prevalence of PTSD in the present study was higher than the previous estimates on PTSD within war and post-conflict communities [1, 2, 32, 33]. One possible reason for this extremely high PTSD prevalence could be the high exposure to war-related traumatic events coupled with the increasing level of violence in the ongoing conflict within DRC. Secondly unlike prior studies in this setting [2], our sample was composed of Congolese refugees that had recently arrived in Nakivale refugee camp due to the increasing violence in their home country. Previous studies in Nakivale refugee camp included refugees from different countries who had stayed in Nakivale refugee settlement for many years [1, 2]..

Consistent with Hypothesis 2, the highest conditional risk of developing PTSD was associated with *being raped*, followed by *witnessing murder of someone* and s*eeing people carrying dead bodies or mutilated bodies* emerged as the third highest conditional risk of developing PTSD. Generally, our findings indicate that both women and men in this sample followed a similar trend in relation to the conditional risk of developing PTSD following specific potentially traumatizing events. Although experiencing rape posed a high conditional risk of developing PTSD within our sample, we also found that *witnessing murder* and *seeing dead bodies* equally posed high levels of conditional risks for developing PTSD. This suggests that in highly traumatized communities, exposure to sexual violence does not singularly pose the risk of developing PTSD in female survivors of war-related violence but goes hand in hand with other traumatic event types, such as murder and seeing dead bodies. This finding is in line with the previous findings of Foa and Tolin [8]. In their review of studies, they observed that both men and women tend to experience different forms of traumatic events and the risk of developing PTSD for women seems to occur across a wide range of traumatic categories including events endorsed by men as well**.** We suggest that the possibility of conditional risks for developing PTSD between men and women be explored further to understand possible confounders and other predicator variables.

The results of this study revealed that the number of potentially traumatizing event types correlated positively with PTSD symptom severity in both male and female refugees. Our results (see also Fig. 1) support our hypothesis 3 that women show a slightly steeper building block effect than men. Concordant with prior research [5, 19, 20], our data thus replicated the building block effect in refugees whereby exposure to different types of traumatic stressors cumulatively increases the risk of developing PTSD symptom severity. Our data thus supports the notion that in circumstances of high levels of trauma exposure, such as in the ongoing conflict within the DRC, there may be no ultimate resilience to ward off PTSD.

Frequent and severe exposure to war-related traumatizing experience may not only heighten the risk of trauma-related disorders, but also seems to increase the risk for other deficits in cognitive domains and psycho-social functioning [19, 27]. The combination of trauma-related symptoms and other mental health problems (PTSD, depression, alcohol abuse, enhanced aggressive behavior) and psycho-social dysfunctioning may contribute to the elevated levels of poverty and lack of opportunities for the future in refugee settings [19, 34]. However, the extremely high prevalence of PTSD for both male and female refugees from DRC calls for the need to implement mental health policies and services geared towards alleviating trauma-related mental illness that affect refugees. Evidence-based research [35] has shown the success of narrative exposure therapy (NET) in treating trauma-related disorders among severely traumatized refugees. In addition to an improvement of trauma-related symptoms, the authors emphasize on the remarkable improvement of psycho-social functioning in everyday life. This is in agreement with previous finding of Hall et al. [38] who found a positive correlation between cognitive processing therapy and social capital among survivors of sexual violence in Eastern DRC. Based on these finding, we argue that the establishment of trauma-focused treatment options in post conflict communities and refugee settlements, would be of great benefit to trauma survivors by improving their mental well-being as well as their overall functioning.

### Limitations

Due to the cross-sectional nature of our study design, our findings do not provide the basis for establishing causal relationships between our variables. Moreover, our sampling technique could have resulted in a selection bias, thus, limiting the generalizability of our findings. Although, the participants talked very openly about their experiences and feelings, our findings could have been subjected to potential biases related to face-to face interviews, to recall due to traumatization (avoidance), or to not disclosing shameful events. Though gender effects have also been reported for DSM-5 PTSD, the findings presented here are based on the PTSD criteria of DSM-IV. In this study, we focused on gender difference in traumatic experiences and PTSD. However, in addition to PTSD symptoms, our highly burdened sample is also very likely to suffer from other mental health problems, such as depressive symptoms, anxiety symptoms or substance abuse. The presented findings refer to PTSD symptom severity only. Finally, we did not assess domestic violence which is known to be another driver of trauma-related illness.

## Conclusions

In conflict and post-conflict communities’ individuals are cumulatively exposed to different potentially traumatizing event types which expose them to a high risk of developing PTSD. Women are at a substantially higher risk of experiencing sexual violence, which in turn seems to have a high conditional risk for PTSD development. We found further evidence for the building block effect that states that cumulative exposure to traumatic event types increases the risk of developing PTSD and that at a certain threshold; everyone exposed to high level of potentially traumatic events ultimately develops PTSD irrespective of gender. Nevertheless, our findings indicate gender differences regarding the building block effect by women presenting higher PTSD symptoms severity than men with low to moderate levels of trauma exposure. One explanation for this gender difference may be the fact that women are more likely to experience sexual violence and that this may pose a continuous rather than a “post”-traumatic stressor. However, this assumption needs to be tested by future research.

## Data Availability

The data sets used and analyzed during the current study are available from the corresponding author on reasonable request.

## Abbreviations

DRC: Democratic Republic of Congo
DSM-IV: Diagnostic and Statistical Manual of Mental Disorders Version 4
PSS-I: Posttraumatic Symptom Scale -Interview
PTSD: Posttraumatic Stress Disorder
